# Impact of digital technology on herders’ grassland leasing-in decisions in Inner Mongolia, China

**DOI:** 10.1371/journal.pone.0331914

**Published:** 2025-09-09

**Authors:** Chen Xue, Mei Yong, Fulin Du, Zhidong Feng, Jiali Han, Haihua Lin

**Affiliations:** 1 College of Economics and Management, Inner Mongolia Agricultural University, Hohhot, China; 2 Faculty of Education, Shinawatra University, Bangtoey, Thailand; 3 College of Finance and Economics, Inner Mongolia Open University, Hohhot, China; Jimma University College of Agriculture and Veterinary Medicine, ETHIOPIA

## Abstract

Against the backdrop of grassland ecological degradation, grassland transfer has become a crucial pathway for optimizing livestock resource allocation and promoting sustainable pastoral development. Based on survey data from 383 herder households in the farming–pastoral ecotone of Inner Mongolia, China, this study applies Heckman models, mediation models, and moderation models to examine the impact of digital technology on herders’ grassland leasing-in decisions and the underlying mechanisms. The results indicate that digital technology significantly increases both the probability and the scale of grassland leasing-in among herders. Moreover, digital technology indirectly promotes grassland leasing-in by enhancing scale efficiency. Notably, strong social networks weaken the facilitating role of digital technology in grassland transfer, whereas weak social networks strengthen it. The impact of digital technology on grassland leasing-in is especially pronounced under environmental regulations with weak incentives but strong constraints. Finally, compared with ordinary herders, digital technology has a significantly positive effect on grassland leasing-in only for new-type operators. In addition, the impact of digital technology is more pronounced among groups with higher levels of education.

## 1. Introduction

Grasslands are among the most widely distributed vegetation types worldwide [1], covering about 40% of the global land area (excluding Antarctica and Greenland) and supporting the livelihoods of nearly one billion people [2]. They perform critical ecological functions, including soil and water conservation, water source retention, and biodiversity protection. Grasslands also act as ecological barriers closely connected to socioeconomic development and regional ecological security [3,4]. They perform critical ecological functions, including soil and water conservation, water source retention, and biodiversity protection. China boasts abundant grassland resources, with its prairie area accounting for approximately 12% of the global total, ranking first in the world [5]. According to data from the Third National Land Survey, grassland area is second only to forest land, making it the second largest land use type in China, covering about 27.56% of the national territory. Consequently, they constitute the largest terrestrial ecosystem in the country [6,7]. However, weak management systems and increasing human disturbances have created severe ecological challenges [8], including shrinking grassland area, declining quality, reduced grazing capacity, and further degradation such as desertification and salinization [9]. Statistics show that nearly 90% of China’s usable natural grasslands have degraded, expanding at a rate of two million hectares annually [10].

Facing these impacts, grassland remains the fundamental resource for herders’ livelihoods. Grassland transfer is a key mechanism for optimizing resource allocation in animal husbandry [11]. Since the reform era, the household contract responsibility system has led to resource misallocation across labor, grassland, livestock, and productive assets [12]. To address resource scarcity and improve production efficiency, the grassland transfer system emerged [13], currently referring mainly to the transfer of grassland management or usage rights [11]. Therefore, in this study, “grassland leasing-in” refers to the practice whereby a herder leases-in grassland from another right holder—via formal contractual arrangements or informal agreements—to acquire grassland management rights. Compared with farming households, herders face more severe resource imbalances, making grassland transfer a vital means to balance resources, break the cycle of degradation and poverty, and promote sustainable development in grassland-based animal husbandry [12].

Herders’ grassland transfer behaviors typically take three forms: non-participation, leasing-in, and leasing-out. In theory, herders engage in land transactions to improve welfare [14]. Leasing-in occurs when herders rent additional grassland to achieve scale economies and higher profits [15], while leasing-out often reflects unstable livestock income and the attraction of stable subsidies, rent, or off-farm employment [12,16]. Non-participation may result from constraints such as limited capital [17–20], ecological concerns [21,22], subsidy policies [23], high transaction costs [24], perceived risks [25], or incomplete markets [19]. A notable emerging constraint is the digital divide, which limits herders’ access to digital tools and platforms that could facilitate transactions. Digital technologies can address these barriers by reducing transaction costs, improving information flow, and enabling off-farm employment [26]. This aligns with broader research on agricultural land transfer, which highlights four mechanisms: reducing transaction costs through digital platforms, enhancing social capital via virtual networks, mitigating information asymmetry, and facilitating labor reallocation to non-agricultural sectors [27,28]. Together, these mechanisms are reshaping traditional patterns of pastoral resource allocation.

Despite this progress, several research gaps remain. First, little attention has been paid to the relationship between digital technology and grassland leasing-in, as most studies focus on farmland transfer. Second, existing analyses emphasize information acquisition, while the efficiency-enhancing role of digital technology remains unexplored. Third, because grassland ecosystems are fragile, grassland transfer is strongly influenced by policy, suggesting that the effect of digital technology may vary across institutional contexts. Fourth, differences in resource endowments among production entities lead to divergent transfer behaviors, but few studies examine how digital technology affects these differences.

To address these gaps, this study focuses on four key questions: What impact does digital technology have on grassland leasing-in? What role does scale efficiency play in this relationship? Does the effect depend on social networks? Does the impact of digital technology vary with external environments and herders’ characteristics? Exploring these questions is crucial for advancing digital rural construction and transforming animal husbandry development models.

## 2. Theoretical basis and hypotheses

### 2.1. Impact of digital technology on grassland leasing-in

According to Schultz’s theory, the introduction of modern production factors is essential for transforming traditional agriculture [29]. As a new production factor, digital technology provides abundant data for pastoral production, streamlines information acquisition [30], enables rapid dissemination and integration of agricultural and pastoral information [31], and supports herders’ grassland leasing-in decisions by improving information availability. This reduces information asymmetry [32,33], lowers information costs, and thereby facilitates grassland transfer. More importantly, digital technology leverages data as a new production factor to exert its enabling function [34]. In animal husbandry, it represents an innovation in production methods, shifting the dominant mode of production from reliance on manual labor to intelligent machinery.

Traditionally, livestock rearing requires extensive manual intervention, which constrains the scale of operations. Theoretically, enabling fewer people to manage larger herds could overcome the main bottleneck to higher output and profitability [35]. Digital technology addresses this constraint by replacing routine, repetitive, and some non-routine tasks, generating a substitution effect [36]. This shift from mechanization to intelligent systems reduces labor intensity and expands the management scope and capacity of each laborer. For example, real-time livestock tracking reduces the time spent locating animals, while remote herd monitoring minimizes unnecessary labor inputs. As a result, with equal or even reduced labor input, herders can manage larger herds, overcome scale bottlenecks, reduce management costs, and enhance scale efficiency. Improved scale efficiency further lowers unit production costs and substantially increases expected returns from expansion. Based on the rational-economic agent assumption, herders are therefore more likely to lease in grassland to expand production and pursue higher returns. However, adopting digital technology also involves risks—such as data tampering, cyberattacks, and unauthorized access—that may undermine operational integrity and reduce expected gains without sufficient safeguards. As Yazdinejad et al. [37] note, smart farming and precision agriculture systems face increasing security threats, requiring robust countermeasures such as encryption, intrusion detection, and secure communication protocols.

Furthermore, while enhancing scale efficiency, digital technology also mitigates livestock disease and management risks, strengthening herders’ confidence in leasing-in and managing grassland. Ultimately, it improves managerial capacity and cost advantages, potentially increasing future demand for additional grassland to exploit scale economies.

Based on this theoretical analysis, the following hypotheses are proposed:

H1:‌ Digital technology positively facilitates herders’ grassland leasing-in behavior.

H2:‌ Digital technology indirectly promotes herders’ grassland leasing-in behavior by enhancing scale efficiency.

### 2.2. Moderating effect of social networks

Social relationships include both strong ties and weak ties. Strong ties usually exist between individuals with frequent interaction and close relationships, often yielding repetitive or redundant information. By contrast, weak ties connect individuals with diverse socio-economic characteristics, such as different experiences, knowledge, and backgrounds, and are generally superior in providing novel, effective, and non-redundant information [38]. A key function of social networks in grassland transfer is information acquisition. Herders obtain policy information and grassland-related details from their social networks. As a non-market pathway, social networks disseminate policy information and help herders understand other stakeholders’ perspectives. The larger the social network, the more diverse the information herders can access [39]. In information-scarce pastoral areas, choosing transaction partners based on personal relationships is the primary way to mitigate risks [40]. The main advantage of strong social networks is their emotional support, which constrains both parties, reduces contract breaches and land degradation, lowers moral hazard and transaction costs, and has historically supported grassland transfer processes [41]. However, with the development of digital technology, the role of strong social networks may be declining. Digital technology reduces information asymmetry, enabling impersonal transactions, and also provides formal contract safeguards, reducing dependence on relational contracts.

Unlike strong ties, weak-tie networks connect groups with different education levels, incomes, and ages, giving them a comparative advantage in accessing non-redundant information. When informal reciprocity cannot meet herders’ demand for grassland resources and expansion, they must seek additional lease information, and the “information bridge” function of weak ties becomes critical [42]. However, transactions in weak-tie networks often require institutional safeguards [43]. Without digital tools, herders with sparse social ties lack institutional support, hindering independent transfers. Conversely, effective use of digital tools can fill these institutional gaps by providing information exchange and formal safeguards, even for sparsely connected herders or transactions between strangers, thereby promoting grassland leasing-in through weak-tie networks. Thus, different social networks may moderate the effect of digital technology on grassland leasing-in in distinct ways. Based on this theoretical analysis, we propose:

H3:‌ Strong social networks weaken, whereas weak social networks strengthen, the positive effect of digital technology on grassland leasing-in.

### 2.3. Heterogeneity in digital technology’s impact on grassland leasing-in

The adoption and diffusion of technology are widely recognized as being shaped by individuals’ educational attainment [44]. Theoretically, herders with higher education generally have stronger abilities in acquiring and processing information, which enables them to better use digital platforms—for example, for online leasing, price comparison, and contract signing. Empirical research supports this view: farmers with higher education show greater willingness to adopt smart agricultural technologies [45]. These cognitive advantages allow them to use digital tools more effectively in practice. By contrast, herders with lower education often face barriers to adopting and applying digital technologies. The higher cognitive costs of understanding tools, operating platforms, or evaluating risks may cause reluctance or mistrust, thereby reducing the utility of these technologies. Consequently, higher education is likely to enhance the positive impact of digital technology on grassland transfer decisions.

At the same time, leasing behaviors are also shaped by other individual characteristics and external policy environments [46–49]. In recent years, China’s livestock production system has shifted from traditional household contracting to new-type operators—such as large-scale households, family ranches, cooperatives, and livestock enterprises—which has accelerated grassland transfer [50]. This is mainly because, first, unlike ordinary herders, new-type operators earn most of their income from livestock production, prioritize long-term development, and typically require large-scale land transfers to meet specialized production needs [51,52]. By contrast, ordinary herders increasingly diversify their livelihoods and engage in pluriactivity [53,54]. According to comparative advantage theory, they reduce labor input in livestock production, focus less on long-term benefits, and prefer to increase non-pastoral income, thereby reducing their likelihood of leasing-in grassland [55,56].

Second, as market-oriented organizations [57], new-type operators have stronger risk-bearing capacity than ordinary herders, who are constrained by subsistence and risk aversion, thereby increasing the probability of leasing-in. Third, higher intensification among new-type operators their adoption of digital technologies, further increasing the probability of leasing-in [58]. Fourth, contiguous operations among new-type operators optimize production efficiency, further increasing incentives for leasing-in.

Moreover, intensified economic activities place substantial pressure on grassland ecosystems [59]. In response, China implemented the Grassland Ecological Compensation Awards (GECA) policy to protect grassland ecology. As the world’s largest ecological compensation program in coverage and beneficiaries, GECA reshapes herders’ expected returns from grasslands and alters their land management strategies [60]. Under grassland degradation, GECA focuses on livestock reduction and grazing bans through incentive and restrictive instruments: restrictive policies impose economic or reputational penalties on non-compliant herders to increase violation costs, while incentive policies provide subsidies to offset losses and externalities from destocking, thereby stabilizing economic expectations.

As a result, herders’ leasing-in behavior shows heterogeneity under different environmental regulations. Specifically, when subsidies fail to cover the opportunity costs of destocking, herders cannot rely on compensation alone, making leasing-in a rational choice for profit. At the same time, strict penalties for overstocking raise the marginal costs of non-compliance, compelling herders to acquire grassland legally to avoid penalties.

Thus, we propose the following hypothesis

H4:‌ The impact of digital technology on grassland leasing-in is heterogeneous.

**Fig 1:**
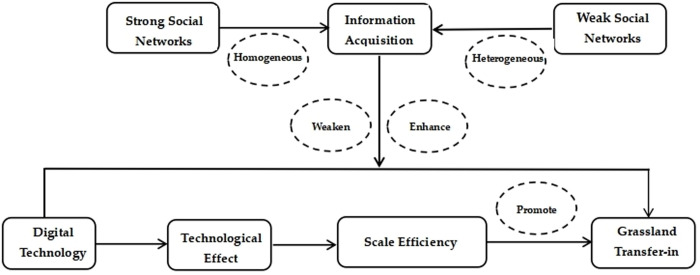
Research framework. Source: Prepared by the authors based on the study’s conceptual design.

This study develops a conceptual framework examining digital technology’s impact on grassland transfer (Fig 1).

## 3. Methodology

### 3.1. Data sources

The data for this study were collected through face-to-face field surveys conducted between May 23 and June 1, 2023, in five pastoral banners of Chifeng City, Inner Mongolia. The study sites lie in the agro-pastoral ecotone of the southern Greater Khingan Mountains and include Keshiketeng, Bairin Right, Bairin Left, Ongniud, and Ar Horqin Banners. Minors were excluded from the survey population. Before data collection, all participants were informed of the research purpose and provided written consent, confirming voluntary participation. The project was approved by the School Research Ethics Panel of the corresponding institution (approval code: NNDKY2024004).

A stratified random sampling design was adopted at the sumu (township) level, covering 31 sampled sumu. Within each stratum, interviewers randomly selected household identifiers from updated administrative rosters of registered herder households provided by local authorities. One adult decision-maker from each household was interviewed face-to-face. The target sample was proportionally allocated across strata according to the number of registered herder households, ensuring representativeness across banners and sumu. After fieldwork, records were screened using a pre-specified protocol. Following rigorous quality checks, 29 questionnaires were excluded due to critical errors, substantial missing values, or extreme outliers that could not be corrected. These excluded cases were distributed across different townships and did not cluster in any specific category of herders, minimizing the risk of systematic bias. The remaining 383 valid questionnaires produced an effective response rate of 93%.

Two primary sources of potential bias are recognized. First, non-response and temporary unavailability during peak husbandry activities may bias the sample toward time-constrained households. This was mitigated by scheduling visits outside peak hours when possible and conducting in-person callbacks. In the empirical analysis, household and operational controls as well as township fixed effects were also included. Second, differences in comprehension, especially among older or less educated respondents, may cause measurement errors. To minimize such errors, trained interviewers explained questionnaire items in detail. Econometric analyses using Heckman and IV-Heckman specifications, along with robustness checks, confirmed that the main results remained robust.

**Fig 2:**
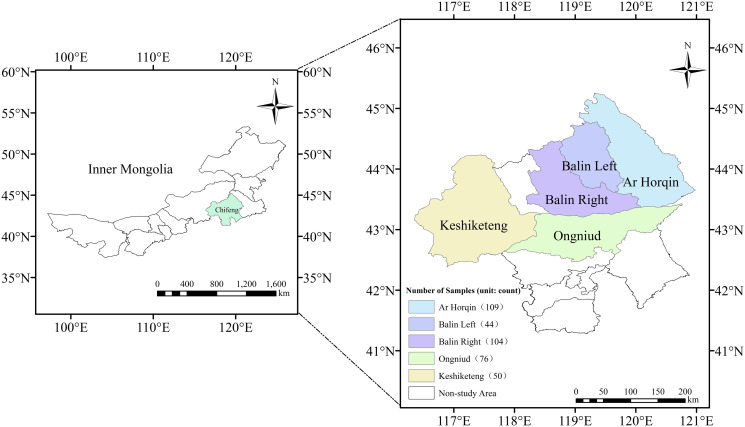
Source of sample data.

This region was selected for two reasons. First, the third national land survey shows that the Inner Mongolia Autonomous Region has about 81.56 million hectares of grassland, accounting for 20.56% of China’s total and 46% of the region’s landmass. Of this, natural pasture covers 71.88 million hectares, artificial pasture 1.91 million hectares, and other types 9.49 million hectares. The grassland ecosystem is highly diverse, with eight major categories, 21 subcategories, and 476 types, forming a rich repository of natural resources. Surveying this area thus provides comprehensive and representative herder samples. Second, as a key agro-pastoral transition zone, it is one of China’s most ecologically vulnerable and poverty-concentrated areas [61,62], making it particularly relevant for studying grassland transfer. The distribution of the survey area is shown in Fig 2.

### 3.2. Model construction

#### 3.2.1. ‌Heckman model.

Grassland leasing-in is a two-stage decision process: first, deciding whether to lease-in grassland, and second, determining the transfer area. Sample selection bias may arise because some herders who wish to lease-in may abstain due to individual constraints. Consequently, only samples that exhibit leasing-in behavior provide observable area data. To address this issue, the Heckman model is employed.

Stage 1: Transfer Participation Decision

A probit regression models herders’ binary transfer decision using fitted values and residuals of digital technology variables alongside exogenous covariates:


$$\text{Probit}({\text{In}}_{\text{i}})=\alpha_{\text{0}}+\alpha_{\text{1}}{\text{Digital}}_{\text{i}}+\alpha_{\text{2}}{\text{Control}}_{\text{i}}+\epsilon_{\text{i}}$$ (1)

where ${\text{In}}_{\text{i}}$ denotes the grassland leasing-in decision (1 = yes; 0 = no), ${\text{Digital}}_{\text{i}}$ represents digital technology variables, ${\text{Control}}_{\text{i}}$ is a vector of control variables, $\alpha_{\text{0}}$ denotes the intercept, $\alpha_{\text{1}}$ and $\alpha_{\text{2}}$ are parameter estimates, and $\epsilon_{\text{i}}$ is the stochastic error term.

Stage 2: Transfer Area Determination

An OLS regression estimates the transfer area using fitted values and residuals of digital technology-related variables with exogenous predictors:


$${\text{Area}}_{\text{i}}=\beta_{\text{0}}+\beta_{\text{1}}{\text{Digital}}_{\text{i}}+\beta_{\text{2}}{\text{Control}}_{\text{i}}+\epsilon_{\text{i}}$$ (2)

where ${\text{Area}}_{\text{i}}$ indicates the grassland leasing-in area, ${\text{Digital}}_{\text{i}}$and ${\text{Control}}_{\text{i}}$are as previously defined, $\beta_{\text{0}}$ is the intercept, $\beta_{\text{1}}$ and $\beta_{\text{2}}$ are parameters, $\epsilon_{\text{i}}$ denotes the error term.

In the Heckman model, technology promotion is used as the identifying variable. It affects herders’ decisions to lease-in grassland but does not directly influence the scale of leasing. The rationale is that agricultural technology extension is a key policy instrument for knowledge dissemination [63]. When organized by local governments, such campaigns change herders’ perceptions [64], and improved cognition is expected to generate more rational behavioral responses [65]. Existing studies also show that cognitive levels significantly influence farmers’ willingness to engage in land transfer [66]. However, without sufficient capital and labor, exposure to technology promotion alone cannot increase the feasible scale of leased grassland. Thus, technical publicity satisfies the relevance conditions for influencing participation in leasing without directly affecting the leased-in area.

Nevertheless, the above estimations may still suffer from endogeneity issues arising from omitted variables, measurement errors, or reverse causality. To address this concern, the present study employs the IV-Heckman model to overcome potential sample endogeneity. Following prior research [67], we use the “digital technology adoption rate within the same sumu (township)” as an instrumental variable for individual adoption. The rationale is twofold: first, the township-level adoption rate is highly correlated with individual adoption, as herders within the same township face similar livestock production environments and share common infrastructure. Second, conditional on control variables, township-level averages do not directly determine an individual household’s leasing scale or decision, which depend on household-specific resources and preferences. Hence, this variable satisfies both the relevance and exogeneity requirements for a valid instrument.

#### 3.2.2. Mediation effect model.

Theoretical analysis suggests that digital technology may indirectly affect grassland leasing-in decisions through the mediating channel of scale efficiency. To test this mechanism empirically, we apply the causal mediation framework developed by Baron and Kenny [68] and extended by Wen [69]. Given the binary nature of the dependent variable, we use the nonparametric bootstrap procedure for mediation testing proposed by Preacher [70], which accommodates dichotomous outcomes without distributional assumptions.


$${{\text{In}}_{\text{i}}=\tau }_{\text{0}}+\tau_{\text{1}}{{\text{Digital}}_{\text{i}}+\tau }_{\text{2}}{\text{Control}}_{\text{i}}+\epsilon_{\text{i}}$$ (3)

$${{\text{SE}}_{\text{i}}=\sigma }_{\text{0}}+\sigma_{\text{1}}{{\text{Digital}}_{\text{i}}+\tau }_{\text{2}}{\text{Control}}_{\text{i}}+\epsilon_{\text{i}}$$ (4)

$${\text{In}}_{\text{i}}^{\prime }=\omega_{\text{0}}+\omega_{\text{1}}{\text{Digital}}_{\text{i}}+{\omega_{\text{2}}{\text{SE}}_{\text{i}}+\omega }_{\text{3}}{\text{Control}}_{\text{i}}+\epsilon_{\text{i}}$$ (5)

where ${\text{SE}}_{\text{i}}$ denote the mediator variable (scale efficiency), ${\text{In}}_{\text{i}}$ denotes the grassland leasing-in decision, ${\text{Digital}}_{\text{i}}$ represents digital technology variables, and ${\text{Control}}_{\text{i}}$ is a vector of control variable.

#### 3.2.3. Moderating effects specification.

To empirically assess the moderating role of social networks, the baseline model is augmented with interaction terms between digital technology and strong or weak social networks. The formal specification is as follows:


$${\text{In}}_{\text{i}}=\varphi_{\text{0}}+\varphi_{\text{1}}{\text{Digital}}_{\text{i}}+{\varphi_{\text{2}}{\text{Social}}_{\text{i}}+\varphi_{\text{3}}{\text{Digital}}_{\text{i}}\times {\text{Social}}_{\text{i}}+\varphi }_{\text{4}}{\text{Control}}_{\text{i}}+\epsilon_{\text{i}}$$ (6)

Here, ${\text{Social}}_{\text{i}}$ denote strong and weak social networks of herder household.

### 3.3. Variable selection

#### 3.3.1. Dependent variable.

Following previous studies [27], grassland transfer decisions are measured using two indicators: (i) a binary variable for leasing-in participation, and (ii) a continuous variable for the acquired grassland area.

#### 3.3.2. Core independent variable.

Digital technology is the primary explanatory variable. Despite improvements in network infrastructure in agro-pastoral regions, a secondary digital divide has emerged. This divide reflects competence gaps in using digital devices, retrieving information, and engaging in online marketing, and has become a major constraint limiting herders’ access to digital benefits [71]. To quantify digital technology use in livestock production, we employ a binary indicator based on herders’ self-reported adoption of digital technologies (e.g., video monitoring, traceability systems, positioning devices, drones, or smart feeding systems). Households that adopt at least one technology are coded as 1, and non-adopters as 0.

#### 3.3.3 Mediating variable.

Scale efficiency is the mediating variable. It is derived from Data Envelopment Analysis (DEA) using DEAP 2.1 software, based on an input-oriented model with variable returns to scale (VRS). Inputs include pastureland (operated grassland area), labor (hired and family labor valued in monetary terms), and capital expenditures (forage, breeding services, fuel, fixed-asset depreciation, and other inputs), consistent with previous studies [72]. The output variable is the total pastoral production value.

#### 3.3.4. Moderating variables.

The moderating variables are strong and weak social networks. Strong ties mainly derive from kinship, close friends, and familiar neighbors, and their maintenance requires substantial gift-giving and ritual obligations [39]. Accordingly, “interpersonal expenditure” is used as the proxy for strong social network [73]. Weak ties lack strong emotional bonds, and “communication expenditure” effectively captures households’ access to broader information [74]. Consistent with prior studies, communication expenditure serves as the proxy for weak social network [75].

#### 3.3.5. Control variables.

The model controls for pastoral household characteristics, operational attributes, and geographic heterogeneity. Individual characteristics encompass household head’s age, health status, education, and political status; household traits include dependency ratio, non-pastoral employment, per capita livestock income, and livestock income share; operational features control for per capita grassland area, per capita livestock scale, barn-feeding duration, and unit production cost. In addition, township-level dummy variables are included to capture regional fixed effects.

### 3.4. Descriptive statistical analysis

Table 1 reports descriptive statistics for the key variables. Overall, about 16% of pastoral households participated in grassland transfer, with an average transferred area of 218 mu. In addition, 39% of households adopted digital technologies in production. For individual characteristics, household heads had an average age of 49 years, generally reported good health, and attained an education level equivalent to junior high school, with only a small proportion serving as village cadres. For household characteristics, families showed relatively low dependency ratios and a clear trend toward non-pastoral livelihoods. Per capita livestock income averaged 55,200 yuan, with livestock husbandry remaining the primary income source. From an operational perspective, households managed an average per capita grassland area of 310 mu and held 96 sheep-equivalent units per capita. Barn-feeding was the predominant rearing method and was associated with higher unit production costs.

**Table 1:** Descriptive statistics and analysis of variables.

Type	Variable	Variable Definition	Mean	Std. Dev.	Min	Max
Dependent Variables	Grassland Leasing-In‌	‌Have pastoral households transferred in grassland? (1 = yes; 0 = no)	0.16	0.37	0.00	1.00
	Grassland Leasing-In Area	Total area of grassland transferred in during the year (in mu, where 1 mu ≈ 666.67 m²)	218.16	852.51	0.00	10000.00
‌Core Explanatory Variable	Digital Technology	Binary indicator of whether households used digital technologies (e.g., video monitoring, traceability systems, positioning devices, drones, or smart feeding systems) in production (1 = yes; 0 = no)	0.39	0.49	0.00	1.00
‌Mediating Variable	Scale Efficiency	Calculated using DEAP2.1 software	0.55	0.26	0.02	1.00
Moderating Variable	Strong Social Network‌	Measured by interpersonal expenditure (in yuan)	15441.25	14560.48	200.00	100000.00
	Weak Social Network	Measured by communication expenditure (in yuan)	3372.47	2584.98	600.00	25000.00
‌Control Variable	Age	Actual age (years)	48.87	9.30	22.00	78.00
	Health Status	1 = good; 2 = minor illness; 3 = serious illness	1.11	0.38	1.00	3.00
	Education	Years of formal schooling	9.06	3.08	0.00	16.00
	Political Status	Whether the household head is a village cadre (1 = yes; 0 = no)	0.21	0.41	0.00	1.00
	Dependency Ratio	(Number of children + elderly)/ Number of working-age members	0.46	0.53	0.00	3.00
	Non-pastoral Employment	Whether any working-age members migrated for off-farm jobs (1 = yes; 0 = no)	0.21	0.41	0.00	1.00
	Per Capita Livestock Income	Annual livestock income per household member (in 10,000 yuan)	5.52	8.33	0.01	77.62
	Livestock Income Share	Livestock income as a percentage of total household income (%)	77.62	27.78	0.17	100.00
	Per Capita Grassland Area	Total grassland managed per capita (mu), calculated as (contracted area + transferred-in area – transferred-out area)	310.46	386.48	0.13	3533.33
	Per Capita Livestock Scale	Year-end livestock inventory per capita (in sheep equivalent units)	95.50	77.73	3.75	630.00
	Barn-feeding Duration	Months per year livestock were confined	7.00	3.02	0.00	12.00
	Unit Production Cost	Cost per sheep equivalent unit (in 10,000 yuan)	0.12	0.22	0.01	4.03
	Regional Dummy	Administrative township (sumu/town) of residence	15.45	9.49	1.00	31.00
Identification Variable	Technology Promotion	Whether households heard government propaganda on new livestock technologies (0 = never; 1 = heard)	0.61	0.49	0.00	1.00

## 4. Empirical analyses

### 4.1. Baseline regression and endogeneity treatment

Table 2 reports the baseline results on the impact of digital technology on grassland leasing-in. In Columns (1) and (2), the coefficients of digital technology are positive and statistically significant at the 10% and 5% levels, respectively. The average marginal effect in Column (1) is 0.0619, implying that households using digital technology are 6% more likely to participate in grassland leasing-in than non-users. Column (2) further shows that adopters lease in about 509 more mu of grassland.

**Table 2:** Estimated results of digital technology on grassland leasing-in.

Variable	Grassland Leasing-In	Grassland Leasing-In Area	Grassland Leasing-In	Grassland Leasing-In Area
	**Heckman (1)**	**Heckman (2)**	**IV-Heckman (3)**	**IV-Heckman (4)**
Digital Technology	0.3140* (0.1765)	508.5991** (230.7832)	2.4042*** (0.6579)	2118.5241** (872.0501)
Age	−0.0179 (0.0114)	−32.9361* (17.5187)	−0.0184 (0.0117)	−23.3133 (16.5767)
Health Status	0.0876 (0.2604)	518.4499 (342.6007)	0.1032 (0.2650)	457.6057 (338.8594)
Education	−0.0488 (0.0314)	−7.8447 (42.5531)	−0.0378 (0.0322)	21.2150 (41.5223)
Political Status	0.4200* (0.2361)	809.9918** (343.5101)	0.4350* (0.2435)	666.6350** (308.3859)
Dependency Ratio	0.2147 (0.1697)	271.9860 (205.2862)	0.0156 (0.1814)	80.7115 (183.6742)
Non-pastoral Employment	−0.1584 (0.2514)	−488.4252 (315.5368)	−0.3634 (0.2672)	−566.5357* (327.9923)
Per Capita Livestock Income	−0.0035 (0.0149)	29.6986 (25.1709)	−0.0075 (0.0154)	33.6806 (25.2176)
Livestock Income Share	0.0001 (0.0036)	−7.4748* (3.8478)	0.0052 (0.0039)	−3.4833 (3.9821)
Per Capita Grassland Area	0.0013*** (0.0003)	3.4229*** (0.4525)	0.0016*** (0.0003)	3.3093*** (0.4144)
Per Capita Livestock Scale	−0.0004 (0.0016)	−3.4799 (2.3650)	−0.0030 (0.0018)	−6.1203** (2.6179)
Barn-feeding Duration	−0.0253 (0.0317)	−127.6525** (38.0100)	−0.0528 (0.0333)	−154.6205*** (42.6831)
Unit Production Cost	−2.6240* (1.5112)	−3166.1370 (2508.9645)	−2.9321* (1.5425)	−2923.2260 (2456.4881)
Regional Dummy	−0.0272** (0.0103)	−16.5619 (14.8164)	−0.0270** (0.0106)	−9.1264 (13.7574)
Technology Promotion	0.3347* (0.1895)		0.4279** (0.1967)	
_cons	0.1048 (0.9111)	192.0669 (1346.3973)	−0.7597 (0.9590)	−426.4596 (1430.8348)
IMR		1460.6482** (637.9280)		1121.7049** (527.7761)
Obs	383	62	383	62
Wald test of exogeneity			11.12***	
First-stage F-statistic			31.9727	

Standard errors in parentheses. *P < 0.1, **P < 0.05, ***P < 0.01.

Columns (3) and (4) address endogeneity from reverse causality and sample selection. Results indicate that the coefficients of digital technologies remain positive and significant. The Wald test is significant at the 1% level, and the first-stage F-statistic is 31.97, confirming instrument validity. This confirms that digital technology promotes grassland leasing-in even after controlling for endogeneity. Hence, Hypothesis 1 is validated: digital technologies significantly increases both the likelihood of grassland leasing-in and the transferred area.

Among personal characteristics, age negatively affects the transferred area. Older households tend to prioritize livelihood stability, and acquiring larger areas involves greater uncertainties and risks. Reduced labor capacity with age also leads households to retain contracted grasslands for subsistence instead of expanding through transfers. Political status exerts a positive effect, since village cadre household heads have greater access and capacity to acquire grassland.

Regarding household characteristics, a higher livestock income share reduces the transferred area. One possible explanation is that more specialized pastoral households typically practice stall-feeding for longer durations, thereby reducing their demand for transferred grassland area.

For operational characteristics, per capita grassland area positively affects transfer-in, whereas confinement feeding duration and unit production costs exert negative effects. Longer confinement reduces grazing demand, while higher costs economically constrain households and limit expansion.

### 4.2. Robustness checks

#### 4.2.1. Alternative estimation methods.

To further address potential sample selection bias, the propensity score matching (PSM) method was employed. As reported in Table 3, the PSM results show that households adopting digital technologies lease in about 123 more units of grassland. These findings confirm the robustness of the baseline estimates.

**Table 3:** PSM estimation results of the effect of digital technology on grassland leasing-in.

Variable	Matching method	ATT	S.E.	T-stat
Grassland Leasing-In Area	Nearest neighbor matching (k = 1)	123.0993*	66.6901	1.85

* *P* < 0.1.

To further validate the reliability of the PSM estimates, a balance test was conducted, and the outcomes are reported in Table 4. As shown in Table 4, most standardized biases were substantially reduced after matching, with all falling below the 20% threshold [76]. Furthermore, post-matching t-tests for all covariates were insignificant, confirming that the assumptions of common support and balance were satisfied. These results further confirm the robustness of the estimates.

**Table 4:** Balance test before and after propensity score matching.

Variable	Unmatched Matched	Mean		%bias	t-test
		Treated	Control		
Age	U	48.404	49.177	−8.4	0.427
	M	48.404	49.47	−11.5	0.323
Health Status	U	1.106	1.1164	−2.8	0.792
	M	1.106	1.1589	−14.2	0.272
Education	U	9.1861	8.9741	6.7	0.511
	M	9.1861	9.0066	5.7	0.626
Political Status	U	0.2450	0.1940	12.3	0.235
	M	0.2450	0.2318	3.2	0.788
Dependency Ratio	U	0.5189	0.4285	16.9	0.105
	M	0.5189	0.5237	−0.9	0.941
Non-pastoral Employment	U	0.2649	0.1810	20.2	0.051
	M	0.2649	0.2318	8.0	0.507
Per Capita Livestock	U	6.5953	4.8208	20.7	0.041
	M	6.5953	6.5545	0.5	0.972
Livestock Income Share	U	73.243	80.464	−25.9	0.013
	M	73.243	72.968	1.0	0.936
Per Capita Grassland Area	U	290.47	323.47	−8.6	0.415
	M	290.47	291.83	−0.4	0.970
Per Capita Livestock Scale	U	106.54	88.31	22.7	0.025
	M	106.54	102.84	4.6	0.717
Barn-feeding Duration	U	7.3775	6.7586	20.9	0.050
	M	7.3775	7.9139	−18.1	0.106
Unit Production Cost	U	0.1376	0.1207	7.0	0.464
	M	0.1376	0.1148	9.4	0.416
Regional Dummy	U	15	15.746	−7.8	0.453
	M	15	15.225	−2.4	0.832
Technology Promotion	U	0.6093	0.6078	0.3	0.976
	M	0.6093	0.5960	2.7	0.815

#### 4.2.2. Alternative dependent variables.

Following prior methodology [77], this study uses grassland rent as an alternative measure of grassland leasing-in and applies a Tobit regression model for robustness testing. The results reported in Table 5 indicate that the coefficients of digital technology remain significantly positive under this specification, thereby reinforcing the robustness of the baseline findings.

**Table 5:** Robustness check: alternative dependent variables.

Variable	Grassland Rent
Digital Technology	3.5650* (1.9993)
Controls	Yes
_cons	2.9834 (10.3409)
‌Obs	383

Standard errors in parentheses. *P < 0.1.

#### 4.2.3. Alternative explanatory variables.

A broader range of digital technology tools adopted by herders indicates a higher level of digital technology adoption. Accordingly, this study replaces the primary explanatory variable with the number of digital technology tool types as an alternative measure and re-estimates the baseline model. The results in Table 6 show that, under this specification, the diversity of digital technology tools has a significantly positive effect on both grassland leasing-in participation and transferred area, thereby reinforcing the robustness of the previous results.

**Table 6:** Robustness check: alternative explanatory variables.

Variable	Grassland Leasing-In (1)	Grassland Leasing-In Area (2)
Digital Technology Tool Types	0.3295** (0.1510)	533.9602** (210.6553)
Controls	Yes	Yes
_cons	0.1068 (0.9105)	19.0272 (1335.1773)
IMR		1591.3835** (644.2673)
‌Obs	383	62

Standard errors in parentheses. **P < 0.05.

#### 4.2.4. Adjusting sample size.

Since a lower proportion of livestock income in total household income indicates reduced reliance on pastoral activities—which may lead to more discretionary grassland leasing decisions [27]—this study conducts a robustness test by excluding households with livestock income shares below 20%. The models and control variables remain unchanged for re-estimation. The results show that, even after sample exclusion, digital technology continues to exert significantly positive effects on both grassland leasing-in participation and transferred area, thereby confirming the robustness of the regression outcomes (Table 7).

**Table 7:** Robustness check: adjusting sample size.

Variable	Grassland Leasing-In (1)	Grassland Leasing-In Area (2)
Digital Technology	0.3800** (0.1803)	518.8180* (279.7783)
Controls	Yes	Yes
_cons	−0.0583 (0.9676)	281.2790 (1562.6672)
IMR		1303.3013* (688.4577)
‌Obs	363	59

Standard errors in parentheses. *P < 0.1, **P < 0.05.

### 4.3. Mediating role of scale efficiency

Table 8 presents the results of the mediation analysis for scale efficiency. The estimates indicate that digital technologies significantly improve scale efficiency at the 1% level, with adoption increasing scale efficiency by approximately 0.07 units relative to non-adopters. Scale efficiency also exerts a significantly positive impact on grassland leasing-in decisions at the 1% level, suggesting that improvements in efficiency substantially increase the likelihood of leasing-in.

**Table 8:** Mediating effects of scale efficiency.

Intermediation effect pathway	a coefficient	b coefficient	Ind _ eff [95% conf. interval]	Dir _ eff [95% conf. interval]	Proportion of total effect%
Digital Technology → Scale Efficiency → Grassland Leasing-In	0.0696*** (0.0225)	0.2456*** (0.0855)	0.0171 [0.0006,0.0336]	0.0482 [-0.0297,0.1261]	26.21%

‌***denotes statistical significance at the 1% level. All control variables are included in the regression but omitted here for brevity. Bootstrap mediation analysis is conducted with 5,000 replications.

The mediation test shows that the confidence interval of the indirect effect excludes zero, whereas that of the direct effect includes zero, confirming a statistically significant indirect effect but an insignificant direct effect. These results confirm that digital technologies influence herders’ leasing-in decisions indirectly through scale efficiency, with the mediation effect accounting for 26.21% of the total effect. The proposed pathway “Digital Technology → Scale Efficiency → Grassland Leasing-In” is empirically supported.

Mechanistically, digital tools substitute for traditional herding methods, thereby reducing management costs and enhancing scale efficiency. Higher scale efficiency allows herders to achieve greater output per unit of input, which in turn incentivizes grassland leasing-in. Overall, these findings provide robust empirical evidence supporting Hypothesis 2.

### 4.4. Moderating effects of social networks

Prior to empirical estimation, the variables of strong and weak social networks were mean-centered to mitigate multicollinearity [78]. Table 9 reports the estimation results for the moderating effects of social networks. The results show that strong social networks have a significantly positive coefficient at the 5% level, whereas their interaction with digital technologies is significantly negative at the same level. This implies that although traditional strong ties enhance the probability of leasing-in, their facilitating role diminishes once herders adopt digital technologies.

**Table 9:** Moderating effects of social networks.

Variable	Grassland Leasing-In	
Digital Technology	0.3522* (0.1800)	0.3231* (0.1872)
Strong Social Network‌	0.0000** (0.0000)	
Digital Technology×Strong Social Network	−0.0000** (0.0000)	
Weak Social Network		−0.0001 (0.0001)
Digital Technology×Weak Social Network		0.0002** (0.0001)
Controls	Yes	Yes
_cons	0.1086 (0.9340)	0.1316 (0.9384)
‌Obs	383	383

Standard errors in parentheses. *P < 0.1, **P < 0.05

By contrast, weak social networks display insignificant direct effects, yet their interaction with digital technologies is significantly positive at the 5% level. This suggests that although weak ties alone do not foster leasing-in decisions, digital technologies unlock their latent value for adopters.

The mechanism operates through two complementary channels: digital technologies enhance herders’ management capacity, while weak social networks supply critical information on leasing opportunities. This technological–informational complementarity generates synergistic effects that substantially facilitate leasing-in decisions. Overall, these findings provide robust empirical support for Hypothesis 3.

### 4.5. Heterogeneity analysis

#### 4.5.1. Heterogeneity by the intensity of environmental regulation.

We further examine whether the impact of digital technologies on grassland leasing-in differs across varying intensities of environmental regulation. To address sample size constraints, households were split into two subsamples—above-mean and below-mean groups—so as to ensure statistical robustness. Following the implementation outcomes of the grassland ecological subsidy and reward policy, herders with grazing ban enforcement intensity above the mean were classified as the “strong-restriction” group, whereas those below the mean comprised the “weak-restriction” group. Likewise, herders with grazing ban incentives above the mean were labeled as the “strong-incentive” group, while those below the mean were categorized as the “weak-incentive” group.

To estimate the probability of leasing-in participation, a Probit regression model was employed, given its suitability for binary dependent variables. As shown in Table 10, digital technologies exert a significantly positive effect on grassland leasing-in within the weak-incentive group. Specifically, column (2) shows that a one-unit increase in digital technology adoption raises the probability of leasing-in participation by 9.50%. In contrast, the effect is statistically insignificant for the strong-incentive group. Likewise, in the strong-restriction group, digital technology adoption exerts a significantly positive effect: column (8) indicates that each additional unit of adoption increases the probability of leasing-in by 9.52%. By contrast, the effect remains insignificant for the weak-restriction group. Taken together, these findings underscore that the effect of digital technologies on leasing-in is conditional on the regulatory context.

**Table 10:** Heterogeneity analysis by intensity of environmental regulation.

Variable	Grassland Leasing-In							
	Incentive-Based Policies				Restriction -Based Policies			
	Weak-incentive Group		Strong-incentive Group		Weak-restriction Group		Strong-restriction Group	
	(1)	(2)	(3)	(4)	(5)	(6)	(7)	(8)
Digital Technology	0.6178* (0.3153)	0.0950** (0.0474)	0.1385 (0.2324)	0.0309 (0.5178)	0.1612 (0.2999)	0.0248 (0.0461)	0.4298* (0.2425)	0.0952* (0.0526)
Controls	Yes	Yes	Yes	Yes	Yes	Yes	Yes	Yes
_cons	−0.0727 (1.7515)		0.2452 (1.1502)		0.0138 (1.5415)		0.9028 (1.2679)	
Obs	173		210		185		198	

Standard errors in parentheses. *P < 0.1, **P < 0.05.

This heterogeneity can be interpreted as follows. Under weak incentives—where subsidies fail to offset economic losses from grazing bans—digital technologies allow herders to expand production by leasing additional grasslands, thereby compensating for subsidy shortfalls. Under strong restrictions, where grassland scarcity is exacerbated, digital technologies assist herders in acquiring legal grazing rights through leasing, thereby enabling compliance while optimizing resource allocation.

#### 4.5.2. Heterogeneity by type of production entity.

We further distinguish between two categories of production entities: new-type operators and ordinary herders. Respondents were classified as new-type operators if they were professional large-scale households, family ranch managers, or members of specialized herders’ cooperatives, while all others were categorized as ordinary herders. Since this study focuses on individual households and data on leading enterprises and agricultural service organizations are scarce, the definition of new-type operators is confined to the three groups mentioned above.

Based on the Probit model, results reported in Table 11 indicate that the coefficient of digital technology adoption for new-type operators is positive and statistically significant at the 5% level. The marginal effect estimates further suggest that a one-unit increase in digital adoption raises the probability of grassland leasing-in by 11%. By contrast, the effect for ordinary herders remains statistically insignificant. These findings highlight the heterogeneous impact of digital technology adoption across different types of production entities.

**Table 11:** Heterogeneity analysis by type of production entity.

Variable	Grassland Leasing-In			
	New-Type Operators		Ordinary Herders	
	Coefficient (1)	Marginal Effect (2)	Coefficient (3)	Marginal Effect (4)
Digital Technology	0.5242** (0.2547)	0.1124** (0.0231)	−0.0170 (0.2910)	−0.0025 (0.0435)
Controls	Yes	Yes	Yes	Yes
_cons	2.2087 (1.3994)		−1.3075 (1.5109)	
‌Obs	181		202	

Standard errors in parentheses. **P < 0.05.

#### 4.5.3. Heterogeneity by educational level.

Since herders with different educational backgrounds vary in their ability to absorb new knowledge, technologies, and ideas, their production decisions may also differ accordingly. Based on the sample characteristics, households with more than nine years of education (above junior high school) were classified as the high-education group, while those with nine years or less (junior high school and below) were defined as the low-education group. The regression results are presented in Table 12. For the high-education group, digital technology adoption exerts a significant positive effect on grassland leasing-in. The marginal effect estimates suggest that a one-unit increase in adoption raises the probability of leasing-in by 11.76%. By contrast, the effect for the low-education group is statistically insignificant. These findings highlight the benefits of digital technology adoption are greater among more educated herders and provide empirical support for Hypothesis 4 (Table 12).

**Table 12:** Heterogeneity analysis by educational level.

Variable	Dependent Variable: Grassland Leasing-In			
	Junior High School and Below		Above Junior High School	
	Coefficient (1)	Marginal Effect (2)	Coefficient (3)	Marginal Effect (4)
Digital Technology	0.3376 (0.2169)	0.0648 (0.0412)	0.7733* (0.4330)	0.1176* (0.0642)
Controls	Yes	Yes	Yes	Yes
_cons	−0.6380 (1.1923)		−0.6875 (2.5765)	
‌Obs	268		115	

Standard errors in parentheses. *P < 0.1.

## 5. Discussion and conclusion

### 5.1. Discussion

This study provides a systematic examination of the multiple pathways through which digital technologies influence herders’ decisions to lease in grassland. The empirical analysis demonstrates that such technologies not only exert a direct positive effect on the likelihood and scale of grassland leasing but also generate indirect benefits through the mediating role of scale efficiency. By enabling herders to overcome constraints on operational expansion and to optimize the allocation of production factors, digital adoption helps break through traditional bottlenecks. The analytical framework also incorporates multidimensional contextual factors—such as social networks, environmental regulations, the type of production entity and educational level—to reveal the multifaceted nature of digital transformation in pastoral regions.

Further evidence suggests that the interaction between digital technologies and social networks is not uniform. In settings characterized by strong social networks, the traditional advantages of these networks tend to weaken in the digital era; conversely, in weaker networks, digital tools can unlock considerable latent value. This finding expands the research perspective on the interplay between social capital and technology adoption, implying that digitalization may reshape the structure of social capital in pastoral communities. The heterogeneous effects associated with environmental regulation further reveal that the influence of digital technologies is more pronounced in contexts where incentives are limited yet regulatory constraints are strong, highlighting the critical role of institutional environments in shaping technological impacts. Moreover, the benefits of digital adoption are found to be concentrated primarily among new types of production entities. This indicates that the digital divide in pastoral areas is rooted less in access to technology per se than in disparities in organizational capacity and specialization—an imbalance that, to some extent, runs counter to the inclusive development goals of current policy.

The contributions of this study are twofold. Theoretically, it develops and empirically validates a “digital technology–scale efficiency–grassland leasing” pathway model, elucidating the moderating roles of social networks and institutional environments, and thereby enriching the research framework linking digital technologies with land transfer processes. Practically, the findings offer targeted guidance for formulating differentiated policies for digital pastoralism: on one hand, technology promotion strategies should be tailored to match varying social network structures and institutional conditions; on the other, policy should focus on narrowing the organizational gap that drives the digital divide, ensuring equitable benefits across diverse production entities through targeted training programs and institutional safeguards.

International experiences provide additional context for interpreting these results. Studies on the Qinghai–Tibet Plateau indicate that digital technologies have been highly effective in grassland monitoring [79], yet their broader development remains dependent on policy frameworks and public investment due to uneven infrastructure provision [80]. In New Zealand, such technologies have created new opportunities to enhance both the sustainability and productivity of grasslands and pastures [81]. In African pastoral areas, the introduction of mobile communication and other digital tools has markedly improved herders’ access to information on forage availability, water resources, and weather conditions [82]—an advantage particularly significant in weakly connected social networks, where it can expand the range of potential transactions. Similarly, research on the European ruminant industry has shown that the adoption of digital technologies is strongly correlated with herd size; compared with small-scale herders, those managing larger herds are more inclined to integrate advanced technologies into their operations [83]. Collectively, these cases reinforce the central conclusion of this study: while digital technologies hold substantial promise for improving grassland resource management, their effectiveness is shaped by social networks, policy support, and the type of production entity.

Limitations remain. The cross-sectional dataset cannot capture dynamic impacts, and leasing-out behavior—often closely linked to leasing-in—is not examined. Future research should use panel data and an integrated framework covering both leasing-in and leasing-out to generate more comprehensive policy insights.

### 5.2. Conclusion

Using micro-level survey data, this study empirically investigates the impact of digital technologies on herders’ grassland leasing-in behavior. The analysis produces four key insights.

First, adopting digital technologies optimizes the allocation of grassland resources and significantly increases both the probability of herders participating in transfer-in and the scale of transferred areas.

Second, digital technologies indirectly enhance the probability of transfer-in by improving scale efficiency, highlighting their role in factor allocation.

Third, strong social networks tend to weaken, whereas weak social networks moderately amplify, the positive influence of digital technologies on transfer-in. This pattern identifies social capital structure as a critical determinant of digital dividends.

Fourth, the impact of digital technology on grassland leasing-in varies with the intensity of environmental regulation and the type of production entity. Specifically, digital adoption has a significant effect on leasing-in under weak-incentive and strong-restriction environments. Moreover, compared with ordinary herders, its positive effect is stronger for those with higher educational attainment and for new-type operators. Taken together, these findings suggest that digital technologies function not as universal enablers but as context-dependent catalysts, with their benefits concentrated where institutional conditions and organizational capacity are most aligned.

## 6. Recommendations

Based on the above analysis, this study proposes the following policy recommendations:

First, promoting inclusive digital technology initiatives is essential. Beyond general advocacy, local governments and industry associations should provide last-mile connectivity subsidies to households in remote pastoral areas, ensuring stable internet access across villages and seasonal camps. This effort can be complemented by distributing “starter digital kits”—such as GPS ear tags, collars, and grass-yield sensors—to low-income herders. At the community level, shared digital service centers with computers, internet access, and trained facilitators should function as hubs for troubleshooting and equipment maintenance. For herders with lower levels of education, governments and local livestock departments should provide regular “one-to-one” or “peer-to-peer” digital literacy training. These programs should emphasize practical skills—such as mobile application use, operation of grassland leasing platforms, online price comparison, and digital contract signing—to help herders overcome barriers to digital adoption.

Second, weak social networks can be leveraged as informational bridges by developing officially endorsed digital platforms for grassland transfer. These platforms should include standardized listing templates, searchable land-availability maps, and secure e-contract modules. To boost weak social networks, these platforms should incorporate open bidding functions and transparent transaction records accessible to all registered users. Trust among unfamiliar users can be strengthened through third-party credit scoring based on transaction history and performance insurance mechanisms that automatically compensate counterparties in case of breach.

Third, the targeted cultivation of digitally enabled new-type operators—such as cooperatives and family ranches—should be prioritized. This requires tailored digital solutions (e.g., automated feed dispensers) together with capacity-building grants linked to measurable adoption outcomes. Subsidized technical advisory visits can support new-type operators in optimizing their operations, while peer-learning networks should diffuse best practices to smaller, traditional herders, thereby lowering technological barriers through spillover effects.

Finally, strengthening the synergy between environmental regulations and digital technologies is essential. In practice, satellite-based grassland monitoring should be combined with real-time alerts to notify herders of grazing limits or degradation risks, enabling compliance before penalties occur. Local authorities should implement compliance-linked incentives, such as reduced land rental fees or direct payments for maintaining stocking rates within carrying capacity thresholds. To ensure fairness, these incentives should be delivered through the same digital platforms used for land transactions, with clear eligibility criteria and transparent, auditable disbursement processes.

## Supporting information

S1 Data Data. (XLSX)
